# Telomere dynamics in a lizard with morph‐specific reproductive investment and self‐maintenance

**DOI:** 10.1002/ece3.2712

**Published:** 2017-06-07

**Authors:** Nicky Rollings, Christopher R. Friesen, Joanna Sudyka, Camilla Whittington, Mathieu Giraudeau, Mark Wilson, Mats Olsson

**Affiliations:** ^1^ School of Biological Sciences University of Sydney Sydney NSW Australia; ^2^ Institute of Environmental Sciences Jagiellonian University Kraków Poland; ^3^ School of Life Sciences Arizona State University Tempe AZ USA; ^4^ Centre for Ecology and Conservation College of Life and Environmental Sciences University of Exeter Penryn UK; ^5^ School of Biological Sciences The University of Wollongong Wollongong Australia; ^6^ Department of Biological and Environmental Sciences University of Gothenburg Gothenburg Sweden

**Keywords:** alternative reproductive tactics, *Ctenophorus pictus*, painted dragon lizard, TA‐65, trade‐offs

## Abstract

Telomeres in human fibroblasts shorten progressively during *in vitro* culturing and trigger replicative senescence. Furthermore, shortened telomeres can be used as biomarkers of disease. These observations have led to the suggestion that telomere dynamics may also be associated with viability and selection for life history variation in non‐human taxa. Model systems to examine this suggestion would particularly benefit from the coexistence of multiple phenotypes within the same species with different life history trade‐offs, since those could be compared in terms of telomere characteristics. This scenario also provokes the classic question of why one morph does not have marginally higher fitness and replaces the others. One explanation is that different morphs have different reproductive tactics with equal relative fitness. In Australian painted dragons (*Ctenophorus pictus*), males differ in head color, the presence or absence of a gular bib, and reproductive expenditure. Red males out‐compete yellow males in dominance contests, while yellow males copulate quickly and have higher success in sperm competition than red males. Males with bibs better defend partners against rival matings, at the cost of loss of body condition. We show that yellow‐headed and bib‐less males have longer telomeres than red, blue and bibbed males, suggesting that telomere length is positively associated with higher investment into self‐maintenance and less reproductive expenditure.

## 1 Introduction

Telomeres are tandem nucleotide repeats (TTAGGG in most metazoans, Gomes, Shay, & Wright, 2010) found at the ends of chromosomes (Blackburn & Gall, 1978; Hug & Lingner, 2006) that can lengthen or shorten during an organism's life in response to stress and ageing (Harley, Futcher, & Greider, 1990; Liu, 2014). These nucleotide repeats are involved in several important functions, such as the triggering of cellular, and possibly, tissue‐ and whole‐organism senescence (von Zglinicki, 2002). Furthermore, telomeres ensure coding DNA is not lost when linear DNA is replicated (the end replication problem, Hug & Lingner, 2006), prevent the accidental fusion of chromosomes by the repair machinery of the cell (through tight binding with proteins, Houben, Moonen, van Schooten, & Hageman, 2008), and are required for the correct alignment and separation of chromosomes during cell division (Lin, Smith, & Blackburn, 2004). These processes are costly and telomeres become shorter over the life of an organism (at least in homeotherms) due to repeated cellular replication and damage caused by reactive oxygen species (ROS, Chen, Hales, & Ozanne, 2007), which is often elevated during stress (Ludlow, Spangenburg, Chin, Cheng, & Roth, 2014). Another contributing factor to the age‐dependent shortening of telomeres may be that up‐regulation of telomerase production carries costs traded off against other self‐maintenance processes. In addition, increased testosterone levels (or reduced levels of estrogen) may increase telomere attrition and explain sex differences in longevity (males have shorter lives, Barrett & Richardson, 2011) because telomere shortening may trigger cellular senescence (Valko et al., 2007). Given the costs and benefits (sometimes substantial) of “long telomeres,” we therefore expect that classic life history trade‐offs are mediated by, or reflected in, telomere dynamics and should vary across morphs with different reproductive tactics.

Balanced polymorphism occurs when individuals within a population exhibit different phenotypes that coexist over evolutionary time. This phenomenon has been an intriguing puzzle in evolutionary ecology for generations (Darwin, 1871; Gross, 1996; Richman, 2000), begging the question why does not one morph (with the marginally higher fitness) replace the others? There are multiple mechanisms that may maintain polymorphism, such as when heterozygous individuals have greater fitness than their homozygous counterparts (Richman, 2000). Alternatively, the relative fitness of a morph may be frequency‐dependent, decreasing as the morph's frequency within a population increases (Gross, 1996). An example of frequency‐dependent selection is the common side‐blotched lizard (*Uta stansburiana*) in which orange‐, blue‐, and yellow‐throated males compete against each other in a classic “rock‐paper‐scissors” game: each morph has a fitness advantage in competition against one morph but is out‐competed by a third morph (Sinervo & Lively, 1996). The highly dominant orange males maintain large territories containing many females but cannot effectively guard these females from sneak copulations by yellow, female mimics (Sinervo & Lively, 1996). Blue males effectively guard against the yellow males but are displaced by orange males. As yellow males specialize in sneaking copulations, they are likely to always face sperm competition and their sperms are under selection for long‐term sperm storage, which results in posthumous paternity (Zamudio & Sinervo, 2000). Interestingly, the aggressive orange males have higher testosterone and shorter lives than yellow sneaker males (Zamudio & Sinervo, 2000).

Phenotypic differences remarkably similar to those of the side‐blotched lizard also occur in the Australian painted dragon lizard (*Ctenophorus pictus*). Males have distinct color‐based strategies with differences in somatic self‐maintenance and reproductive expenditure, which predicts among‐morph differences in telomere attrition without having to account for phylogenetic differences associated with among‐species comparisons of telomere dynamics (since the morphs, largely, share the same genome). Male *C*. *pictus* are head and gular color‐polymorphic, with individuals having red, orange, yellow, or no (“blue”) head coloration. All males have blue coloration on their body sides. Most research has focused on the red and yellow morphs, since orange and blue morphs have only started appearing in the population in 1997. The yellow coloration is carotenoid‐based, while the red is likely pteridine‐based (Olsson, Healey, Wapstra et al., 2007). Red males emerge earlier post‐hibernation (Olsson, Healey, Wapstra et al., 2007), experience a greater increase in testosterone throughout the day than yellow males, and outcompete yellow males in dominance contests (Healey, Uller, & Olsson, 2007; Olsson, Healey, & Astheimer, 2007). Conversely, yellow males have larger testes and four times higher reproductive success in sperm competition trials than red males, despite much shorter copulations (Olsson, Schwartz, Uller, & Healey, 2009). This suggests that these two morphs exhibit alternative reproductive tactics that likely result from fundamental differences in trade‐offs of resources between testes, testosterone‐driven aggression, and longevity. Additionally, male dragons possess another polymorphic feature: a yellow, probably carotenoid‐based, gular bib (Healey & Olsson, 2009). The presence of a bib identifies more short‐term reproductively successful males, producing more single paternity clutches, at a substantially higher loss in body condition due to mate defense than bib‐less males (Olsson, Healey, Wapstra, & Uller, 2009). Thus, polymorphisms could offer, for the first time, explanations of intraspecific variation in telomeres among individuals that share most of their genome but differ in reproductive tactics and expenditure. The variation in reproduction and self‐maintenance trade‐offs among the morphs is likely to stem from among‐morph differences at a cellular level, and manifest in differences in age‐ and growth‐related telomeres.

In order to better understand the underlying role of telomeres in the mediation of life history biology, we used a two‐pronged approach. Given their differences in reproductive expenditure, somatic maintenance, growth, ageing, stress, and elevated testosterone levels, telomere dynamics are predicted to vary among morphs. We therefore compared telomere lengths among morphs of *C. pictus*. We also attempted to directly manipulate telomere length through an increase in telomerase activity using TA‐65, a herbal supplement that is purported to function as a telomerase activator (Bernardes de Jesus et al., 2011) by up‐regulating telomerase transcription factors which bind to the activation domain of the telomerase gene and up‐regulate transcription (Valko et al., 2007). This manipulation unfortunately had no effect and is reported in Appendix I to minimize the interruption of the text.

## 2 Methods

**Figure 1:**
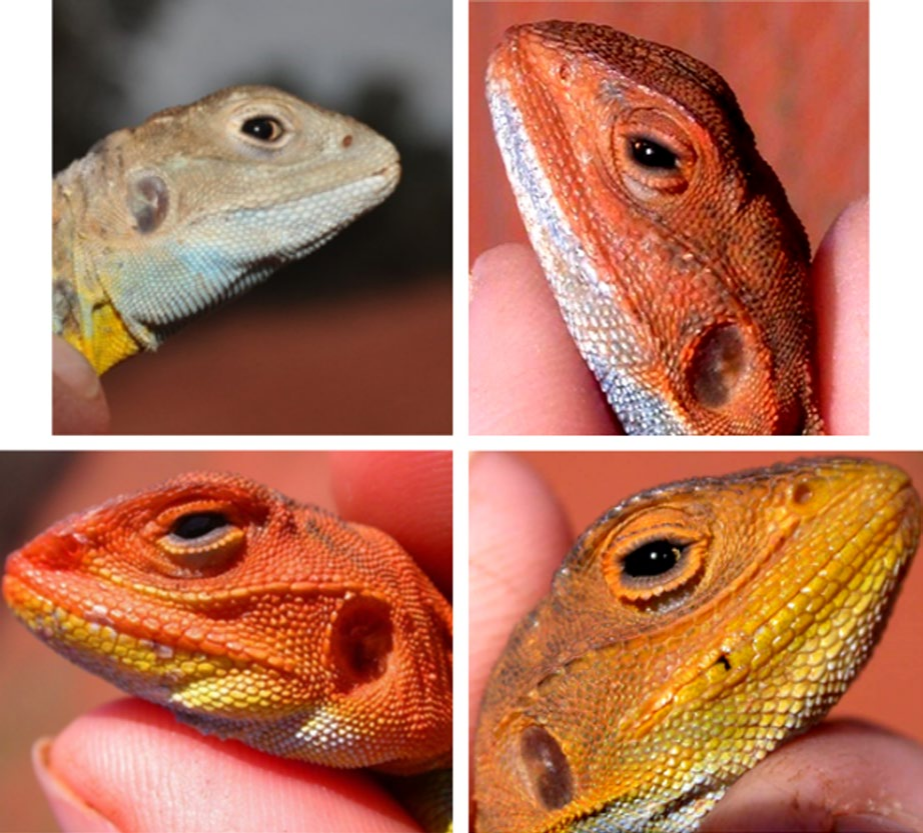
Plates of the four different male painted dragon morphs (*Ctenophorus pictus*)

We quantified the telomere lengths of mature male painted dragons (*Ctenophorus pictus*) of four different head color morphs (Figure 1). The dragons are ideal models for this research as they are short‐lived and age rapidly (surviving ~1 year in the wild, Olsson, Healey, Wapstra et al., 2007), making it more likely that attempts to manipulate telomeres will be detected (Olsson, Tobler, Healey, Perrin, & Wilson, 2012).

This work was performed under the Animal Ethics permit 2013/6050 at the University of Sydney. Mature (~9 months old) male lizards were caught by noose or hand at Yathong Nature Reserve, NSW, Australia (145°35′E; 32°35′S) and taken to holding facilities at the University of Sydney in October 2014 where they were housed for the duration of the experiments. Adult males were housed individually in opaque plastic tubs (330 × 520 × 360 mm) with sandy substrate and exposed to a 12‐h light: 12‐h dark light cycle. The males were housed in three different rooms for logistical reasons, but morphs were randomly distributed among rooms. The lizards were fed mealworms and crickets, dusted with calcium and multivitamins, to satiation every day and the cages were misted with water once a day. Heat lamps and ceramic hides were provided to allow the lizards to thermoregulate to their preferred body temperature (36°C; M.O., unpublished data obtained from cloacal temperature readings in the wild).

### 2.1 Blood sampling

Blood (~150 μl) was sampled using a capillary tube prior to and after the completion of the treatment by rupturing the *vena angularis* (in the corner of the mouth) with the tip of a syringe. Collected blood was mixed with heparin and centrifuged, the plasma removed and the remaining cells resuspended with 200 μl of PBS. One milliliter of RNAlater (Sigma Aldrich, Australia) was added and the diluted blood cell solution for qPCR stored immediately at −80°C.

### 2.2 Quantifying telomere length: qPCR

To analyze telomere length, we first purified DNA from 50 μl of the using a DNeasy Blood and Tissue Kit (Qiagen, Australia), according to the manufacturer's instructions. RNase A (Qiagen, Australia) was added at the recommended concentration. The DNA concentration (ng/μl) of each sample was measured in duplicate using a Pherastar FS (BMG, Labtech, Germany) and aliquots diluted to 10 ng/μl using the AE buffer provided in the DNA extraction kit. Samples were then stored at −30°C. Telomere length was measured using real‐time quantitative PCR (qPCR) using SensiMix SYBR No‐ROX Kit (Bioline, Sydney, Australia). The telomere primers used were Telb1 (5′‐CGGTTTGTTTGGGTTTGGGTTTGGGTTTGGGTTTGGGTT‐3′) and Telb2 (5′‐GGCTTGCCTTACCCTTACCCTTACCCTTACCCTTACCCT‐3′) (Criscuolo et al., 2009). The 18S ribosomal RNA (18S) gene (92 bp amplicon in *Anolis*) was selected as the reference gene as it had previously been validated in a reptile (Plot, Criscuolo, Zahn, & Georges, 2012). The primer sequences used were 18S‐F (5′‐GAGGTGAAATTCTTGGACCGG‐3′) and 18S‐R (5′‐CGAACCTCCGACTTTCGTTCT‐3′). The melt curves produced for both telomere and 18S after amplification by qPCR displayed a single peak, indicating specific amplification of the DNA sequence. The qPCR was performed in a final volume of 20 μl for both telomeres and 18S. DNA of 10 ng was used per reaction, and the primers were used at a concentration of 250 nM. SensiMix SYBR No‐ROX Master Mix (Bioline, Australia) of 11.25 μl was added per reaction and MgCl_2_ was added for a reaction concentration of 1.7 mM. Reactions were run in triplicate for each sample. Amplifications were carried out in a Rotor‐Gene 6000 thermocycler (Qiagen, Australia) using an initial Taq activation step at 95°C for 10 min, and a total of 40 cycles of 95°C for 15 s, 60°C for 15 s and 72°C for 15 s. A melt curve was created after each run over the temperature range of 60–95°C to ensure no non‐specific product amplification. No‐template control reactions were run in triplicate for each primer set during every qPCR run to ensure no contamination. Standard curves were created, using the blood of a randomly selected lizard, for both telomeres and 18S to ensure consistent rates of amplification over a wide range of DNA concentrations. The reaction was considered consistent when a straight line with an *R* ^2^ exceeding .985 could be fitted to the values obtained. Threefold serial dilutions were created, starting at a concentration of 26.67 ng/μl down to 0.037 ng/μl, with seven different concentrations in total (Supplemental Figure S1) giving a linear dynamic range of 0.037–26.67 ng/μl. The efficiency of the telomere amplification was 1.05 and the efficiency of the 18S amplification was 0.96. All samples fell within the concentration range generated by the standard curve. In all runs, the no‐template controls had a Cq value at least 10 times higher than the lowest sample measured, indicating that contaminant DNA made up a maximum of approximately 0.001% of the original DNA concentration. LinRegPCR 2015.2 (Ruijter et al., 2009; Tuomi, Voorbraak, Jones, & Ruijter, 2010) was used to analyze the qPCR data. LinRegPCR calculates individual starting concentrations based on the average efficiency of an amplicon, the baseline fluorescence, and the threshold cycle values. The starting concentrations of telomere (T) and control gene (S; 18S) were used to determine the relative telomere length with the calculation T/S. Telomeres and the control gene were assessed in separate runs. The mean inter‐assay coefficients of variation for qPCR runs for telomere (*n *=* *13) and 18S (*n *=* *13) amplification were 16.73% and 38.21%, respectively, calculated using a reference sample at 10 ng/μl that was included in all runs. Due to the high inter‐assay coefficient for the 18S runs, we checked individual values and found an outlier which inflated the inter‐assay coefficient. When this outlier was removed, the mean inter‐assay coefficient was 23.52%. To test whether this value was more reliable, we calculated a new mean inter‐assay coefficient based on a standard from the standard curve that was included in all runs at a concentration of 8.89 ng/μl. Under these conditions, the mean inter‐assay coefficient was 22.30%. This suggests that the actual mean inter‐assay coefficient for 18S was approximately 23%. The mean (±*SD*) intra‐assay coefficients of variation for telomere and 18S runs were 14.63 ± 0.1% and 12.52 ± 0.09%, respectively. Mean (±*SD*) amplification efficiencies generated by LinRegPCR across telomere and 18S qPCR runs were 1.89 ± 0.04 and 1.98 ± 0.01, respectively. LinRegPCR efficiency values can be compared with the efficiency obtained by a standard curve by subtracting 1. The difference in efficiency between the standard curve and LinRegPCR method is likely due to the manual setting of the Cq in the standard curve method.

### 2.3 Statistical analysis

For analysis of morph‐specific effects on telomere length, the qPCR data were first entered into a mixed model analysis in Proc MIXED SAS 9.4 (SAS Institute) using telomere length in February (end date of experiment) as the response variable, the predictor fixed factors morph (yellow, orange, red, and blue), and bib (bibbed and not bibbed), and with the room in which a lizard was held (numbered 1–3) as a random factor. When “room” was not significant in a likelihood ratio test (χ^2^ < 1, *p *>* *.9), we performed the corresponding analysis in Proc GLM (in order to obtain *R* ^2^ values for our analyses). Telomere length in December was used as a covariate, which controlled telomere length at the onset of the experimental period. This also constrains the analysis to non‐interstitial telomeres, since it effectively measures the change in telomere length (under the assumption that interstitial telomeres do not change during the experimental period). That said, we do not suggest that interstitial telomeres are irrelevant to viability and other fitness effects under selection in association with telomere dynamics. The data obtained from this experiment will be archived in Dryad.

## 3 Results

**Figure 2:**
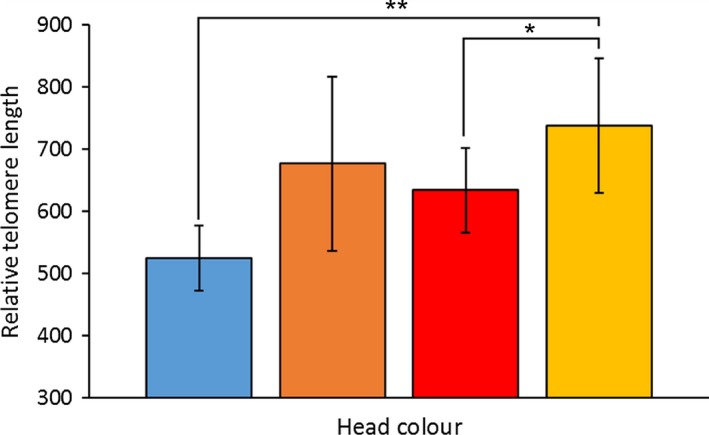
Mean (±*SE*) relative telomere lengths (RTL) of the different morphs from blood sampled in February. RTL of yellow males was significantly higher than red and blue males (see Table 1)

**Figure 3:**
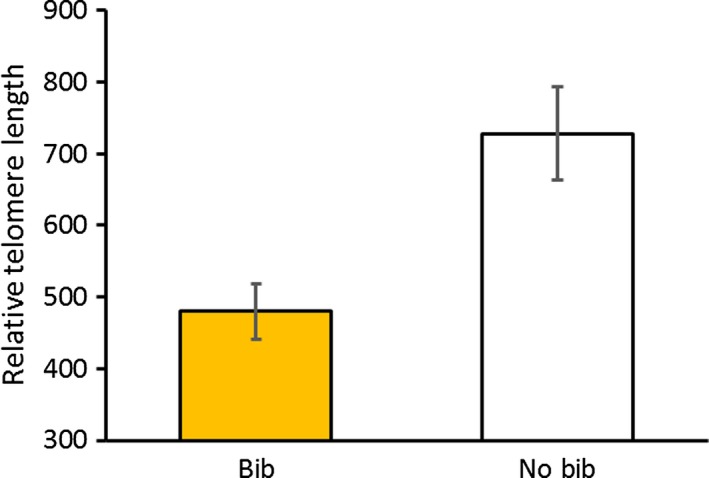
Mean (±*SE*) relative telomere lengths of males with and without bibs in blood sampled in February (at the end of the experiment). Bibbed males had significantly shorter telomeres than males without bibs (*p *=* *.0096)

In accordance with predictions, yellow‐headed males (*n *=* *27) had telomeres that were significantly longer than both red (*n *=* *20) and blue males (*n *=* *17, Table 1 and Figure 2), whereas orange‐headed males (*n *=* *9) were intermediate between yellow and red males, as previously demonstrated for some condition‐dependent traits in this species (Healey & Olsson, 2009). Our prediction that bibbed males (*n *=* *24) with more costly reproductive investments should have shorter telomeres was also upheld; males without bibs (*n *=* *52) had telomeres 1.5 times longer than those of bibbed males (Figure 3). There was no significant interaction between head color and presence of bib. Telomere length in February was also significantly predicted by telomere length at the onset of the experiment in December (Table 1).

**Table 1:** GLM analysis of the interactions of telomeres in February with morph type (blue, orange, red, and yellow) and bib presence (bib and no bib) in painted dragons, *Ctenophorus pictus*. Telomere length in December (TelDec) was used as a covariate

Source	*df*	Sum of squares	Mean square	*F* value	Pr > *F*
Model	5	36.22747047	7.24549409	20.42	<0.0001
Error	67	23.77252953	0.35481387		
Corrected Total	72	60.00000000			

*R* ^2^	Coeff Var	Root *MSE*	TelFeb Mean
.603791	6.11974E17	0.595663	9.7335E‐17

Source	*df*	Type I SS	Mean square	*F* Value	Pr > *F*
Morph	3	5.33457552	1.77819184	5.01	0.0034
Bib	1	4.00673348	4.00673348	11.29	0.0013
TelDec	1	26.88616147	26.88616147	75.78	<0.0001

Source	*df*	Type III SS	Mean square	*F* Value	Pr > *F*
Morph	3	3.84154096	1.28051365	3.61	0.0177
Bib	1	2.52779438	2.52779438	7.12	0.0095
TelDec	1	26.88616147	26.88616147	75.78	<0.0001

Parameter	Estimate	Standard error	*t* Value	Pr > |*t*|
Intercept	−0.0361405961	0.15521276	−0.23	0.8166
Morph BLUE	−0.5469063510	0.18497301	−2.96	0.0043
Morph ORANGE	−0.0918655966	0.23245313	−0.40	0.6940
Morph RED	−0.4033722194	0.17964460	−2.25	0.0280
Morph YELLOW	0.0000000000	–	–	–
Bib NO	0.4165981249	0.15607981	2.67	0.0095
Bib YES	0.0000000000	–	–	–
TelDec	0.6868996062	0.07890948	8.70	<0.0001

## 4 Discussion

This study provides the first evidence that individuals with different reproductive and life history tactics of the same species have different telomere dynamics. Red and yellow males have well‐established strategies described in our previous work, which provides context for the morph‐dependent telomere attrition. The telomeres of red males were shorter than those of yellow males, potentially revealing a cost associated with red males' higher levels of testosterone and earlier reproductive activities in spring, such as territory establishment. In yellow males, on the other hand, we would predict lower testosterone‐dependent metabolic rate and lower ROS levels, resulting in less telomere attrition. The shorter telomeres of the bibbed males provided additional support for the hypothesis that cellular maintenance is traded off against costs of greater, short‐term reproductive success (Olsson, Healey et al., 2009) and that the phenotypic trade‐off between these traits differ among morphs.

The results obtained for the orange and blue males are intriguing but, as their reproductive behavior and strategies have not been explicitly investigated, the implications of their telomere dynamics are less straightforward. While not significantly different, the telomere length of the orange males was numerically intermediate to the red and yellow males, suggesting that orange males are heterozygotes of red and yellow alleles. Although previous research indicates that the system of color inheritance is more complex (Olsson, Healey, Wapstra et al., 2007), the life history strategy of orange males may still be some intermediate to that of red and yellow males. Blue males had the shortest telomeres and this may indicate higher levels of ROS (perhaps as a result of lower levels of antioxidants). It is tempting to speculate that pigments (e.g., carotenoids) are used to counter damaging ROS effects, leading to the lack of head coloration observed in blue males. However, blue males are still capable of producing bibs. While the exact composition of the yellow bib pigment is unknown, it may contain both carotenoids and pteridines, indicating that ROS levels are not sufficiently high to remove all pigment‐based coloration. Furthermore, this interpretation is further complicated by our experimental demonstration that carotenoid intake has no depressing effect on ROS levels (Olsson, Wilson, Isaksson, Uller, & Mott, 2008). In order to understand the mechanisms behind the telomere dynamics of these two morphs, additional studies of their life history and reproductive strategies are required.

To conclude, our results demonstrate that telomeres, polymorphisms, and life history strategies are strongly interlinked. We have observed differences in telomere dynamics across not just one, but two different types of polymorphisms within a single species, revealing telomeres to capture the trade‐offs at a cellular level caused by alternative reproductive tactics.

## Conflict of Interest

The authors have no conflict of interest to declare.

## Appendix I

### I.1

Based on the results in Reichert et al. (2014), we predicted that TA‐65 would have telomere‐enhancing effects across all morphs and that telomeres would be shorter in high‐investment strategies (thus predicting relatively shorter telomeres in red and bibbed morphs). We therefore assessed whether TA‐65 had the capacity to alter telomere length, possibly through the amount of telomerase activity and telomere length. Given its oral administration, this would be a non‐invasive, direct manipulation of telomere length and its repercussions (Bernardes de Jesus et al., 2011; Reichert et al., 2014).

Males were weighed to the nearest 0.01 g and snout‐to‐vent and total length were measured to the nearest 1.0 mm prior to commencing the TA‐65 treatment. In addition, head color and presence of a bib were recorded and the males were randomly assigned to the TA‐65 treatment (*n *=* *41) or control (*n *=* *35) groups. Males were treated each weekday for 7 weeks, across January and February, with 0.7 mg of TA‐65 (T.A. Sciences, USA), dissolved in 100 μl of saline, given orally. This dosage was selected as an increase in telomere length was observed when zebra finches were treated with 0.5 mg per 100 μl of water (Reichert et al., 2014). The dosage was increased by 40% to account for the decrease in body temperature and metabolic rate experienced by reptiles at night. The TA‐65 solution was prepared each day just prior to administration. Control males were treated identically but given only saline.

TA‐65 did not significantly affect telomere length (*p *>* *.40 in all analyses) and was backwards‐eliminated from all analyses reported on in this manuscript.

Future studies of telomere dynamics should ideally include telomere manipulation experiments. Although telomerase knock‐out organisms have been a successful tool for studying telomere dynamics (Anchelin et al., 2013; Mourkioti et al., 2013), the difficulty and cost of generating knock‐outs limit our ability to investigate telomeres in non‐model organisms. A compound that could be orally administered would be ideal for these purposes; however, our experiments showed no effect of TA‐65 on lizard telomere length. There are a few possible explanations for this: (1) Typical telomerase activity in painted dragons may maintain telomeres at their maximum stable length for each morph; thus, higher telomerase activity may not increase telomeres further. (2) Previous studies have focused on endotherms and thus the dosage chosen may not have been appropriate for an ectotherm (although in the current experiment the bird dose (Reichert et al., 2014) was increased by 40% to compensate for the fall in nightly body temperature to ambient levels in an ectotherm). (3) The telomerase activator needs to be able to bind to the activation domain of the telomerase gene, and if the activation domain and telomerase activator are not appropriately matched, binding may not occur, nullifying the desired manipulation. Only future dose–response effects across a wider range of concentrations will clarify the utility of this compound for potential future telomere manipulation work in ectotherms.
