# Supplementary material for: Telomere dynamics in a lizard with morph‐specific reproductive investment and self‐maintenance
Source: Ecol Evol. 2017 Jun 7;7(14):5163–9. doi: 10.1002/ece3.2712 (PMC5528203; doi:10.1002/ece3.2712)
Supplement: Supplementary file 1 [file ECE3-7-5163-s001.docx]

Figure S1: Standard curves were generated to determine the consistency of the qPCR reactions over a wide range of concentrations. Diamonds represent the reference gene 18S and squares represent telomeres.
